# Understanding in the Australian aged care sector of reablement interventions for people living with dementia: a qualitative content analysis

**DOI:** 10.1186/s12913-020-4977-1

**Published:** 2020-02-24

**Authors:** Claire M. C. O’Connor, Meredith Gresham, Roslyn G. Poulos, Lindy Clemson, Katherine S. McGilton, Ian D. Cameron, Wendy Hudson, Helen Radoslovich, Joan Jackman, Christopher J. Poulos

**Affiliations:** 1 Centre for Positive Ageing, HammondCare, 4 Spicer Ave, Hammondville, NSW 2170 Australia; 2 School of Public Health and Community Medicine, University of New South Wales, Sydney, NSW Australia; 3 Dementia Centre, HammondCare, Greenwich, NSW Australia; 4 Faculty of Health Sciences, University of Sydney, Sydney, NSW Australia; 5 University of Toronto, Toronto, Canada; 6 John Walsh Centre for Rehabilitation Research, Faculty of Medicine and Health, University of Sydney, Sydney, NSW Australia; 7 Brightwater Care Group, Perth, WA Australia; 8 Helping Hand Aged Care, Adelaide, SA Australia; 9 Dementia Australia, Sydney, NSW Australia

**Keywords:** Function, Dementia, Cognitive impairment, Service sector, Activities of daily living, Qualitative content analysis, Reablement

## Abstract

**Background:**

Reablement has potential for enhancing function and independence in people with dementia. In order to enhance the use of evidence-based reablement in this population, this study sought to understand the current practices and needs of the sector around these interventions.

**Methods:**

A purposive sample of 22 Australian aged and community-care providers participated in a semi-structured interview. Qualitative content analysis was applied to the data, with key themes interpreted within the context of the study aims: to explore (1) what reablement interventions are currently being offered to people living with dementia in Australia, and (2) what are key factors that will contribute to enhanced uptake of reablement interventions in dementia practice.

**Results:**

Four themes emerged: (1) ‘what reablement interventions are being offered’, outlined a range of exercise and cognitive/social interventions, with only a proportion generated from a clear evidence-base, (2) ‘what’s in a name’, illustrated the range of terms used to describe reablement, (3) ‘whose role is it’, highlighted the confusion around the range of health professionals involved in providing reablement interventions, and (4) ‘perceived barriers and enablers to providing reablement to people living with dementia’, described a range of factors that both hinder and support current reablement practice.

**Conclusions:**

Reablement interventions currently provided for people living with dementia in Australia are variable, with confusion around the definition of reablement, and apparently limited use of evidence-informed interventions. A multifaceted approach involving an evidence-informed and freely-accessible resource, and taking into account the varied levels of influence within the aged care sector would support uptake and implementation of reablement interventions for people living with dementia.

## Background

It is estimated that nearly one million people will be living with dementia in Australia by 2050 [1]. A leading cause of disability, dementia is projected to cost around A$83 billion in health services by the 2060s [2]. The provision of effective, evidence-based interventions is paramount in addressing disability and reducing costs associated with dementia. There is currently an Australian Government focus on ‘reablement’ and maximising or improving function in people living with dementia in the community [3]. While research (which is cited within government guidelines, Commonwealth of Australia, [3]) has defined reablement as an approach that promotes the regaining or maintenance of functional performance in older people [4], much of it specifically excludes people with dementia.

Reablement shares features, such as maintaining or improving functional ability, with a number of related concepts including rehabilitation and restorative care. However, reablement has been described as a less intensive approach that has potential for enhancing function and independence in people with mild-moderate dementia [3]. As the degenerative course of dementia is yet to be halted, reablement for people living with dementia refers to an approach that aims to maintain function as much as possible, regain lost function wherever possible, and adapt and compensate for functional changes [5, 6]. Evidence to support reablement approaches for people living with dementia is growing [7, 8], yet gaps in the knowledge remain, for example, understanding around models of dementia community care involving reablement [5]. Conceptualising research within and dynamically across the social, physical and policy environment is critical to effective knowledge translation [9]. For reablement in dementia, a key area yet to be explored is knowledge on reablement and related approaches from within the community care sector. Qualitative data on this influential factor would complement the growing trial-based body of evidence and identify specific gaps to address for effective translation of reablement to practice.

The recently released Cognitive Decline Partnership Centre (*CDPC) Clinical Practice Guidelines and Principles of Care for People with Dementia* [10] provide an important advance in critically evaluating the evidence for interventions that could delay the onset of functional decline, or improve functioning and quality of life, for people with dementia. Operationalising these guidelines for the community and residential aged care sectors to enhance the use of evidence-based reablement interventions for people with mild to moderate dementia is the logical next step. In order to do this it is necessary to first understand the current practices and needs of the sector around access to and delivery of reablement interventions for people living with dementia.

Therefore, the aims of this study are to explore what reablement interventions are currently being offered to people living with dementia in Australia, and what are key factors that will contribute to enhanced uptake of reablement interventions in dementia practice. These aims will be achieved through determining the aged and community-care sector’s understanding of reablement (and related concepts) in the context of the person living with dementia, and what service providers perceive as barriers or enablers to the use of reablement in practice.

## Methods

### Participants

A purposive sample of aged and community-care providers from across New South Wales, South Australia, and Western Australia was recruited (*n* = 22). Inclusion criteria for providers were that they must: (1) provide services to people who are living with mild-moderate dementia (as these individuals are most likely to be able to participate in reablement interventions), (2) fit within the study sampling frame (Table 1), and (3) have one or more members of staff identified by the provider as being willing to participate in the interview and able to authoritatively comment on their dementia services. Residential aged care and community aged care organisations were both included, however the sample was weighted towards community aged care providers as these organisations were more likely to service people living with mild-moderate dementia. In order to achieve broad representation of the industry [11, 12], the sampling frame for included organisations was stratified across four factors: geographic location, dementia diagnosis, organisational model, and organisation size (see Table 1 for a description). Further, to ensure a richer contribution to the data, interviewees with a range of perspectives on the industry were sought e.g. managers, allied health, and dementia-specialists. Providers listed on the Australian Government’s *My Aged Care* website (https://www.myagedcare.gov.au/) were identified with the assistance of CDPC partner organisations and contacted to determine their interest in participating in an interview. Once interest was obtained from an organisation, an appropriate interviewee was nominated to participate in the interview. The interviewee was sent a participant information sheet and a copy of the verbal consent form. At the scheduled interview, verbal consent was established to conduct and audio record the interview. Recruitment continued until saturation was reached and no new themes emerged from the data [11, 12]. The study was approved by the University of New South Wales Human Research ethics committee.

**Table 1:** Stratification factors applied to interview sample of aged and community-care providers

Stratification factor	Definition and (annotation) used to identify interview quotes
Geographic location	Regional/remote (R), metro (M), and providers with both regional/remote and metro sites (R/M)
Diagnosis	Providers offering dementia-specific services (D) vs general aged care providers (G)
Organisational model	Not-for-profit (NFP), for-profit (P), and Government funded (Gov) business models
Size	Small (S) providers operating within a single region e.g. one service within one city, and large (L) providers operating within multiple regions e.g. multiple sites across different cities and or states
Interviewee role	Managers (Mx), allied health (AH), and managers with a background in allied health (Mx/AH)

Twenty-two interviews were conducted in total. The role of interviewees within their organisation ranged across managers (45%), allied health professionals (23%) and management with a background in allied health (32%). Within these roles, there were also people who identified as program coordinators (14%), and/or having a dementia-specific role (18%). Three interviews involved two interviewees representing a single provider (quotations denoted using ‘ID-no. a/b’). In these instances, interviewees worked collaboratively to respond to interview questions. The majority of interviewees were working for a range of not-for-profit care providers (82%) that were operating within their own Australian state (73%). Almost half (45%) of the providers offered solely community services, while the remainder also offered residential aged care. For further details on participating providers, see Table 2.

**Table 2:** Overview of interviewee/service provider details (*n* = 22)

Role of interviewee within organisation	Management	10
	Allied health	5
	Management with allied health background	7
Organisational business model	Not-for-profit	18
	For profit	2
	Government	2
Number of Australian states provider operates in	1 state	16
	2–3 states	4
	> 3 states	2
Location of sites	Metro	8
	Regional	2
	Metro & regional	12
Residential care facilities	0	10
	1–10	7
	> 10	5
Residential care places (i.e. bed numbers)	0	10
	1–250	2
	251–1000	4
	> 1000	5
	DK	1
Number of Australian Government-funded Home Care Packages delivered	0	6
	1–500	10
	> 500	5
	DK	1
Other funded community services delivered	CHSP	12
	HACC	5
	Other e.g. privately funded	4
	DK	1
Service focus	General aged care	6
	Dementia specific	15
	All adults	1
Interviewee-estimated percent of clients with dementia within service	0–30%	3
	31–50%	6
	> 50%	5
	DK	8

*CHSP* Commonwealth Home Support Program, *DK* interviewee did not know the information requested, *HACC* Home and Community Care, *Dementia specific* includes services within organisation specifically for people living with dementia

### Study procedure

Interviews were conducted between May – July 2017 by the first author (CMCOC) via telephone, which has been highlighted as an effective method for conducting semi-structured interviews [13]; one interview was conducted face-to-face. Interviewees were provided with an overview of the interview questions prior to the scheduled interview (Additional file 1: Supplement 1). The semi-structured interview was generated in consultation with the project investigator team in a collaborative process. Questions were intended to address the aims of the study by elucidating participant understanding around the concept of reablement for people living with dementia, and to gain an understanding of current practice in that area. Questions were open-ended, and respondents were given the opportunity to add further information they felt was relevant. Interviews were digitally recorded and lasted on average 52 min (30–90 min).

### Epistemology

Qualitative content analysis was conducted within a “factist” perspective on the assumption that the interview data provided a relatively accurate representation of the Australian service sector [14, 15]. Adhering to the sampling frame supported this theoretical position of analysis, which was used to reflect the reality of aged care provider understandings through semantic themes developed around the specified research questions. The analysis progressed from describing the sematic themes identified in the interview data, summarising these into key thematic categories, and finally, interpreting these findings within the context of the study aims [16].

### Data analysis and credibility

Analysis began during the interviews where the interviewer (CMCOC) used clarifying statements and questions in a bid to clarify any ambiguous responses, ensuring accuracy of recorded data [17]. Interviews were audio-recorded and transcribed verbatim by an external transcription service. All transcripts were checked against the recordings for accuracy and clarified where possible. All data were read by CMCOC, and a process of independent auditing was conducted, with 13% (*n* = 3) of the interviews also analysed by MG, a researcher experienced in qualitative methodology. Any disparities were clarified in person through in-depth discussion. Throughout the analytic process, a detailed audit trail was maintained [18]. This included: the research proposal, interview development, sampling frame development, data collection (both audio-recordings and transcribed raw data), data analysis (including notes and diagrams), theme development, and framework development.

Qualitative content analysis was used as an evidence-based approach that allowed for the deductive and inductive systematic interpretation and classification of the interview-generated text data [19, 20]. Preliminary analysis involved a deductive approach whereby categories were developed based around the explicit content of participant responses to interview questions [15]. Therefore, data analysis was initially guided by the focused semi-structured interview questions, but beyond this, was not influenced by a priori models [20, 21]. An inductive approach of open coding was then instigated to identify patterns within these pre-identified categories, but also across the data set as a whole. Transcripts were initially read to gain a general understanding of the data. Detailed readings of the transcripts were then undertaken, using a step by step process of summarising, coding, reduction, condensation, exact checking and revising the data [20, 21]. This cycle was repeated until sufficient reliability and accuracy of the coding was achieved [22]. If new codes were identified during the analysis, any previously analysed transcripts were re-analysed. Through a process of abstraction, data ultimately emerged into categories and themes that supported understanding around the research questions [19, 23]. Participant stratification factors were considered as a comparator during analysis to identify any relevant differences in the findings e.g. manager versus allied health. Credibility of the findings was supported by including direct quotations from the transcribed text to illustrate themes [21]. De-identified quotations (ID-no.) were annotated according to interviewee role and where that respondent fit within the stratification matrix (Table 1). The final step in the analysis process was to develop a framework to highlight the interplay between the identified key categories and themes.

## Results

**Fig. 1:**
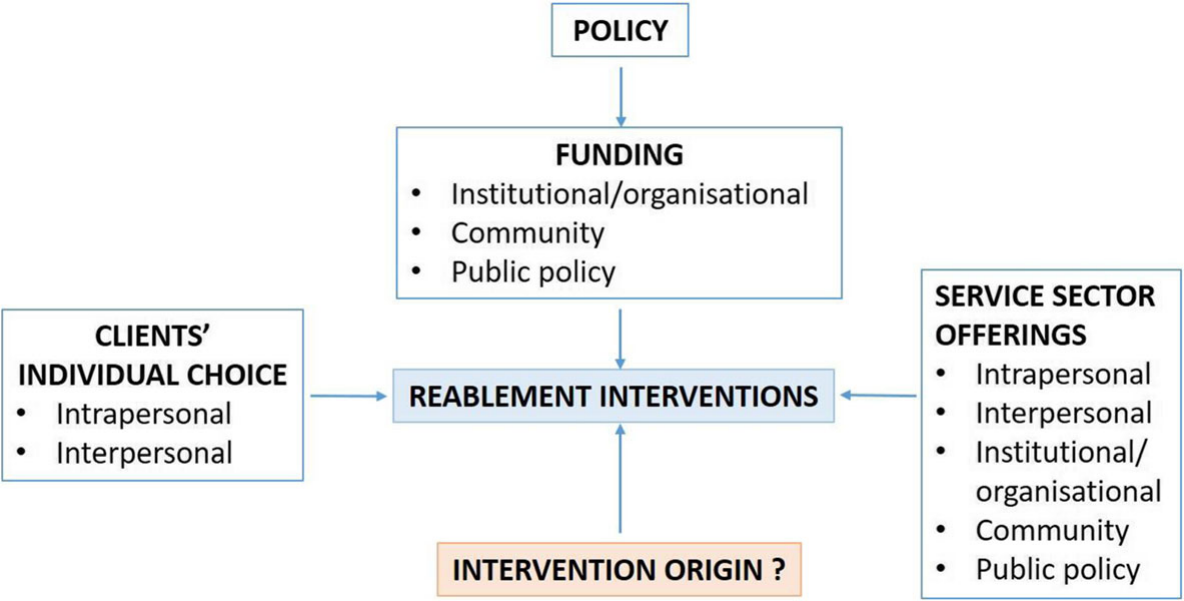
Factorial interplay impacting reablement programs offered to people living with dementia (with mapped socio-ecological model constructs). Figure 1 frames the interplay between the key categories and themes generated from the interviews. Reablement programs that are offered appear to be driven by a number of factors, including Government policy and the associated funding models that have outlined reablement as a focus for good practice in aged care [3]. Each individual care organisation then decides which programs will be offered within their service. The programs that are ultimately taken up are ostensibly dependent on the individual choice of clients when using their assigned funding packages. The remaining question following these interviews is, where are the majority of currently offered reablement interventions coming from?

The main findings from the interview data presented below offer a framework of factors contributing to the use of reablement interventions for people living with dementia within the aged-care sector (Fig. 1). Results are thematically arranged around the research aims, based on the interview schedule and qualitative content analysis.

### Aim 1: what reablement interventions are currently being offered to people living with dementia in Australia?

This aim was addressed through three themes generated from the data. Primarily, participants outlined the exercise and cognitive and social reablement programs being offered. Second, the range of terms used to describe reablement services being offered was discussed, and third, participant’s understanding of different staff roles within these services was explored.

### What reablement interventions are being offered?

Interviews revealed that a range of reablement interventions are being offered to both people with and without dementia. There were no clear differences in the overall programs provided by small vs large organisations; each offered a range of overlapping programs and strategies that fell into two broad categories: exercise programs and cognitive and social programs.

#### Exercise

Of the interventions involving exercise, only a few organisations reported using evidence-based programs (and these were mostly for fall prevention) such as the LiFE functional exercise program [24], Stepping On fall prevention program [25], and the Otago exercise program [26, 27].

*“we’re trying to adapt some of those programs, so the Otago, probably basing a lot of, well I know my work personally I’m basing a lot of it on that program because it can be delivered I guess in different ways as well, and it just gives a good outline of the exercises that we use to get some improvement or to reduce the incidence of falls” (ID-17; AH,DS,L,R/M,NFP).*

*“the LiFE program which is an exercise program based on daily activity … so building into someone’s daily activities … around falls prevention … we’d also use the Otago as well” (ID-8; Mx/AH,G,S,M,NFP).*

The majority of participants discussed using exercise but were vague regarding whether the programs were based on research protocols. For example, participants made reference to home exercise programs, exercise classes, falls and mobility, having an on-site gym, tai-chi, Pilates and yoga, and walking programs.

*“in the community the carers have been through an exercise program, so if they’re out there doing the cleaning they’ll be getting the person they’re doing the cleaning for to help them as part of an exercise routine” (ID-2b; MX,DS,S,M,NFP).*

*“rather than do formal exercise we’ll do things that mean we’re doing bowling or bowls or something that means people have got to get up and walk around and use, get out of their chairs basically … and we dance. So it’s more about informal exercise rather than formal exercise” (ID-10; Mx/AH,DS,S,M,Gov).*

#### Cognitive and social interventions

Of the cognitive and social programs, cognitive stimulation therapy [28, 29] and using a Montessori approach [30] were the only programs discussed that may have been generated from an evidence-based protocol. However it remained unclear if each organisation that reported using these approaches were actually following the research protocol or if they had developed their own protocol.

*“we have the cognitive stimulation program … they’re not big groups … we have clients who … have indicated for example just off the top of my head have indicated a real passion for cars. So then that group will come together and it will be like a discussion group but the focus will be a series of cards to trigger their memory around oh, what was this car, does anyone remember what this car was, oh what did it involve? Did you ever drive one like that? So it’s about triggering their memory and encouraging, maximising their memory around their passion” (ID-6; Mx/AH,DS,S,M,NFP).*

*“They have run particular programmes around cognitive stimulation and the staff have all been trained in maintenance for that as well, they use a Montessori approach in the daily programming” (ID-20a; Mx/AH,G,S,M,Gov).*

A range of other cognitive and social programs were reported across the interviews, however the origin of these remained unclear. Identified programs and activities included good/positive thinking groups, arts programs, music programs, activities programs, outings groups, lifestyle programs, cooking and gardening, class groups and games, active minds and wellbeing groups, structured social activities, and a memory support unit. There was a lack of programs specifically addressing everyday functional ability and independence. References to these outcomes were more general and often intertwined with comments around exercise or cognitive and social programs, or limited to discussion around the role of occupational therapy.

*“trying to build that functional capacity as well by improving people’s strength and balance” (ID-17; AH,DS,L,R/M,NFP).*

*“assisted technology … establishment of very clear routines, and providing that person with the necessary equipment that they might need” (ID-13; AH,G,S,R,P).*

There was a general lack of clarity around the basis of a majority of the reablement interventions that participants reported as being on offer in their services.

### What’s in a name?

A similar understanding around a range of specific terms being used in the sector was expressed by both allied health and management. The most common term participants reported using in their organisation that related to regaining or maintaining functional performance in people with dementia was ‘reablement’, followed by ‘restorative care’, ‘wellness’, ‘rehabilitation’, and ‘functional ability’. The terms being used were driven by different factors, including: government policy, such as documents that guide home support services [3], the geographic location of the organisation, and the values that respondents placed on the terms. For instance, a term may not be seen as relevant to a particular service, or may be associated with a negative connotation.

*“With the changes in the aged care reforms, and the new guidelines, reablement and wellness have almost become buzz words” (ID-20a; Mx/AH,G,S,M,Gov).*

*“I don’t think we’ve done that much on restorative care per se” (ID-14; MX,G,S,M,NFP).*

*“rehab to a certain extent has got a negative connotation because you talk about rehab, people think about drugs and alcohol rehab”. (ID-2b*; *Mx,DS,S,M,NFP).*

Overall, there was confusion around the understanding and meaning of these terms, with participants offering variable definitions and citing difficulty in differentiating between terms.

*“people are still trying to get their heads around what these terms mean, and how they apply in their community” (ID-11; Mx,G,S,R,NFP).*

*“a lot of the stuff we do we call it wellness, we branch it under wellness a lot but when you break it down I can see that it was restorative or reablement or it was a different approach” (ID-18; Mx/AH,DS,L,R/M,NFP).*

*“it’s so difficult, reablement, restorative care, rehabilitation, they’re kind of all, they’ve become quite interchangeable I think” (ID-20b; Mx/AH,G,S,M,Gov).*

Despite a range of terms being used, participants identified general similarities across the approaches, with particular focus of building on intrinsic capacity and maximising function.

### Whose role is it?

The role of different professional groups in reablement interventions supporting people with dementia to maintain or improve their functional ability and independence was explored. Respondents expressed similar understanding around the roles of different health professionals. Key staff in the maintenance or improvement of functional ability and independence were identified as occupational therapists, whose goal was highlighted as keeping people independent and at home, and physiotherapists, whose goal was understood to be around physical function.

*“I don’t know how you can do best practice dementia care without an OT [occupational therapist] personally” (ID-7; AH,DS,L,R/M,NFP).*

*“it’s a bit like a car, the physiotherapist is the mechanic who repairs the car but the occupational therapist is the driver, test drives it to ensure that the repairs have been done” (ID-6; Mx/AH,DS,S,M,NFP).*

In contrast, exercise physiologists were less commonly used across the organisations, and there tended to be some confusion around the difference between their role and physiotherapy. There was some uncertainty as to the roles of other members on the team in general. For example, when asked about what occupational therapy programs were available in their organisation, there was uncertainty from both management *“I don’t know them. I’m not that close to the on-the-ground staff in that sense” (ID-12; Mx,DS,L,R/M,NFP)*, and from other clinicians *“I really don’t even know what half the people in the health care team do” (ID-21b; AH,DS,L,R/M,NFP)*. A number of interviewees indicated they believe an overlap exists between all of the health roles. For example, in regards to home assessments, one respondent stated that *“the physios can do some of that as well” (ID-11; Mx,G,S,R,NFP)*, while another reported that their exercise classes were *“run by an OT” (ID-18; Mx/AH,DS,L,R/M,NFP)*.

When asked about other professions that may have an important role in maintaining or improving function in people living with dementia, a range of other roles were identified. The most frequently cited across the interviews were care support workers, who were seen as crucial to providing consistent, on-the-ground support for people living with dementia. The role of support workers in implementing reablement interventions in collaboration with allied health professionals was also highlighted.

*“our carers out in the community, they’ve got a really important role because they see our members [ie. clients] every day … recognising change in our members, recognise decline or recognising areas that they need support in” (ID-18; Mx/AH,DS,L,R/M,NFP).*

*“if there’s an active program there’s usually an OT component to it, but certainly the maintenance is the program staff doing that” (ID-20a; Mx/AH,G,S,M,Gov).*

Management was also seen as important in terms of planning client services and to *“provide leadership to the care staff so that they can embrace the reablement and restorative rehab model” (ID-6; Mx/AH,DS,S,M,NFP)*. Other roles identified as playing an important role in reablement for people living with dementia were: nurses, therapy assistants, podiatrists, speech pathologists, social workers, dieticians, psychologists, and general practitioners or geriatricians.

### Aim 2: what are key factors that will contribute to enhanced uptake of reablement interventions in dementia practice

Aim two was addressed through one over-arching theme exploring the perceived barriers and enablers to providing reablement to people living with dementia. Participants described a range of factors that both hinder and support current reablement practice.

### Perceived barriers to providing reablement to people living with dementia

#### Funding

Participants saw a number of factors contributing as barriers to implementing more reablement interventions for people living with dementia. The most frequently cited barrier was around funding. Participants felt that there was *“a lack of* [government funded] *packages” (ID-7; AH,DS,L,R/M,NFP)*, that funding was not flexible enough to allow for increased time and unique challenges that arise when working with people living with dementia, and there was no funding allocated for appropriate training of care staff in delivering reablement interventions.

*“part of the issue is time and money because rather than spending half an hour doing a quick shower with somebody, you’ve got to be there for an hour building the relationship and of course you have to pay for an hour’s care rather than half an hour’s care” (ID-21a; AH,DS,L,R/M,NFP).*

For people with dementia residing in aged care facilities, the current Australian residential funding model was viewed as a disincentive to delivering reablement interventions. Participants cited that the Government funding model “*is based on dependence” (ID-2a; Mx,DS,S,M,NFP)* and *“doesn’t actually encourage reablement” (ID-6; Mx/AH,DS,S,M,NFP)*. Within the community sector, the new Consumer Directed Care model that provides consumers with greater control over their personally allocated funding to access services and care (Ottman et al. 2013) was reported as difficult to navigate and that *“people are so confused by the system” (ID-11; Mx,G,S,R,NFP),* with clients unsure of how they could use their allocated funds.

#### Stigma

Barriers to clients accessing reablement interventions were reported as being broader than just being related to confusion around funding. Participants reported that *“stigma of dementia” (ID-1; Mx,DS,L,R/M,NFP)* and the *“initial fear by clients and cares about accessing services to support them” (ID-5; Mx,DS,L,R/M,NFP)* was felt amongst people living in the community, and that was limiting their access to reablement interventions that may support their function.

#### Organisational limitations

From within organisations, barriers to implementation of reablement interventions were identified as having staff not specifically trained in dementia care, high rates of staff turnover, and issues associated with geographical remoteness. While the majority of services in the sample were in metropolitan areas, or were headquartered in metropolitan areas with some regional/remote branches, two organisations were entirely located in regional/remote areas. The different barriers which arose for these services were around long *“wait time” (ID-1; Mx,DS,L,R/M,NFP)* for services, or economies of scale, such as *“you have people who live out of town, so geographical distance to do that visit …*.” *(ID-13; AH,G,S,R,P)* or

*“programs are generally pitched to a certain number of people attending … what happens if you only have two people, you know, how do you make it pay … the reality is they’re probably not going to get it” (ID-11; Mx,G,S,R,NFP)*.

#### External limitations

Finally, challenges were raised around working with external referring organisations, such as the Regional Assessment Service (RAS) and general practitioners who were cited as having limitations *“in terms of knowing that these services we provide would be suitable as a reablement type service for someone with mild to moderate dementia” (ID-5; Mx,DS,L,R/M,NFP)*. Further, greater de-regulation and competition within the aged care sector, such as the new Consumer Directed Care model, was described as having negatively impacted inter-organisational collaborations.

*“the collaboration is not there … it’s become more competitive, and so there’s not as much … the willingness to work with each other is not as great as what it used to be” (ID-6; Mx/AH,DS,S,M,NFP)*.

### Perceived enablers to providing reablement to people living with dementia

#### Organisational support

In contrast, participants also discussed factors they saw as enabling, or supportive to their provision of reablement interventions to people living with dementia. Within organisations, having *“an organisation that is committed” (ID-18; Mx/AH,DS,L,R/M,NFP)* from the top-down to providing reablement interventions to clients with dementia, management that provides leadership in the reablement approach and care workers who are supported in their approach to care was seen as important.

*“trust in your staff with the management to be able to react to the things that they say and do, and the outcomes that they celebrate … when the support worker comes back with really good reablement or wellness initiatives, we celebrate that too and share it with other staff members who also get, I suppose, buoyed from the fact that they’re much more valued out there as support workers” (ID-4; Mx,G,S,M,NFP)*.

#### Offering dementia-specific services

Initially there were some differences in opinion around the provision of dementia-specific versus general services for people living with dementia. Some respondents commented that offering the same approach for all clients was best, e.g. *“Whether you’ve got dementia or not is not an issue” (ID-11; Mx,G,S,R,NFP)*, and *“I don’t like to label people with dementia … they are an independent person that needs support” (ID-4; Mx,G,S,M,NFP)*.

However, when later asked what factors would support the provision of services to promote functional ability in people living with dementia, the concept of more dementia-specific services and approaches was highlighted as important. Key factors identified included adapting programs or the approach to care to fit with the needs of people living with dementia, having a dementia-specific focus from the organisational perspective, and having staff specifically trained in dementia.

*“ensure that we adapt the programs so we will deliver services that are appropriate for people with cognitive loss and for people with dementia” (ID-8; Mx/AH,G,S,M,NFP).*

*“right from the top; so the board are committed, the CEOs are committed to being a dementia-friendly organisation” (ID-18; Mx/AH,DS,L,R/M,NFP).*

*“having a nurse practitioner of dementia care to champion it a bit more in our organisation is a really positive way forward” (ID-5; Mx,DS L,R/M,NFP).*

Following these identified benefits, a number (*n* = 9) of both large and small dementia-specific organisations reported to *“provide training to our care workers and [/or] all of our staff” (ID-3; Mx,DS,L,R/M,NFP)* on dementia.

#### Skilled staff

Having a team environment with skilled staff working together was also seen as supportive; particularly when allied health staff worked closely with the support workers, and when an organisation had dementia-specific staff roles to guide the team in their approach to services.

*“taken the OT out of the office and onto the floor to be mentoring and validating and valuing of staff who are doing the frontline care in role modelling individual as care into practice” (ID-7; AH,DS,L,R/M,NFP)*.

Participants also raised strategies they employ that support them in providing reablement interventions. These included working in collaboration and *“partnerships with families” (ID-7; AH,DS,L,R/M,NFP)*, employing *“thought outside the square” (ID-1; Mx,DS,L,R/M,NFP)* and flexibility around the way they provide their services, and ensuring to *“work with our staff” (ID-18; Mx/AH,DS,L,R/M,NFP)* for support, training and engagement.

#### External factors

A number of supporting factors that were external to their organisation were also discussed. These included recommending clients take advantage of externally run programs and services, such as free community groups, with one respondent commenting *“I don’t even think there is a cost to those, so they’re wonderful” (ID-3; Mx,DS,L,R/M,NFP)*, and external expertise such as a *“dementia link support worker” (ID-11; Mx,G,S,R,NFP)*. Many of the participant’s organisations were taking advantage of the education services and resources provided by the various Dementia Australia (formerly Alzheimer’s Australia) chapters. Finally, some organisations were implementing alternative approaches to providing more reablement interventions, such as *“collaboration with other organisations …*. *organisations linking into our strengths, and visa versa” (ID-6; Mx/AH,DS,S,M,NFP)*, or working with research teams.

*“our research and development unit is really good at trying to run programs with students … so that’s a big facilitator for us where we can have students, student-led programs or even research programs that are helping our clients to deliver more services” (ID-17; AH,DS,L,R/M,NFP)*.

Overall, a range of barriers and enablers to providing reablement interventions for people living with dementia were discussed, highlighting the complex interplay of contributing factors to effective delivery.

## Discussion

This paper presents the results from a series of interviews investigating (1) what reablement interventions are currently being offered to people living with dementia in Australia, and (2) what the key factors are that will contribute to enhanced uptake of reablement interventions in dementia practice. The broad sample of providers interviewed contributed to a representative overview of the Australian aged care sector. Interviews revealed a lack of clarity around the use of evidence-based reablement practice to support function for people with dementia. Therefore, there is a risk that people with dementia are being offered ‘reablement’ interventions that may not be based on evidence, suggesting that government bodies and individuals may be spending funds on potentially ineffective reablement interventions. As illustrated in Fig. 1, our findings highlight the complex interaction of factors impacting on the provision of these approaches within the current care environment. These identified levels of influence align with the five constructs outlined in the socio-ecological model of health promotion (intrapersonal, interpersonal, institutional/organisational, community, and public policy), which provided a useful guide to interpret the findings in the following discussion [9, 31]. Applying these frameworks in combination facilitated a more holistic view, with our study framework illustrating links between the varying levels of the socio-ecological model [32].

Interviews involved a diverse group of professionals to ensure a range of intrapersonal attributes such as prior knowledge, skills and attitudes. Prior to the interview an overview of questions was provided, to allow participants to consider concepts such as reablement before being interviewed. Despite this, and despite government policy [3] and funding promoting the use of reablement in community practice, there remained a lot of confusion around reablement and related concepts. This highlights the need for increased clarity around this field of practice to ensure mutual understanding across the sector. There remains no consistent definition of reablement in the literature, which undoubtedly contributes to confusion in the sector. Reablement involves maximising intrinsic capacity and using environmental modifiers to maintain or improve an individual’s functional ability [4], and describes a continuum of services that can include restorative care and rehabilitation [5]. Because of this range of what reablement entails, it follows that different health professionals will be appropriate depending on the types of services required by each client. The development of a universal definition of reablement will be an important step forward in effectively translating research into practice. In parallel with professionals, this intrapersonal factor also encompasses the person living with the dementia and their family, who are at the centre of these reablement interventions and ultimately will be making decisions around which interventions are taken up. Education must therefore, extend to the broader community to dispel stigmas and provide information around positive approaches towards supporting people living with dementia [33, 34].

The unique interpersonal challenges involved with working with people living with dementia, and that this is not appropriately reflected in funding arrangements was discussed. For example, group situations are not always suitable for a person with dementia, and interventions with this population often require tailoring based on the individual’s remaining abilities [35], which may take more time and additional resources. This echoes previous work that health professionals need more time when working in this field [36], and extends beyond funding where family members often play an integral role in supporting engagement in reablement interventions [37]. Additionally, there was confusion around the different roles of health professionals, such as whether allied health professionals and/or care support workers should be developing and delivering reablement interventions to clients with dementia. The provision of good quality care can be negatively affected by poor inter-professional collaborations [38]. Moving forward, the sustainability of reablement interventions may be enhanced by involving both allied health and support workers in a collaborative, multidisciplinary model of care [39].

Organisational support from the top-down was discussed as a positive feature to providing reablement interventions to people living with dementia. Structures from within the organisation such as supportive management and training programs to ensure all staff are skilled and encouraged to practice within a reablement framework were also raised as beneficial, concepts reflected in previous research [40]. Despite this, limitations regarding lack of sufficient funding to support this were also identified. Across the organisations, a range of reablement interventions for people living with dementia were discussed, but it was unclear where the majority of these programs originated. This resonates with previous research identifying a variety of barriers to the use of evidence-based practice amongst health professionals [41, 42], such as lack of organisational support for staff to maintain engagement with current best practice evidence [42, 43]. There exists a pressing need to develop evidence-informed and freely-accessible resources that are carefully formatted to provide clear information on appropriate intervention approaches to better support people living with dementia. In parallel, the introduction of trained facilitators is one approach that has been identified as having potential to enhance the implementation of novel healthcare approaches such as reablement interventions into practice [44].

The community from both within and between organisations was perceived to impact on the provision of reablement interventions for people with dementia. The importance of collaborative relationships between the various professionals within organisations is a notion that continues to appear throughout the layers in this interpretation using the social-ecological model, reflecting previous research [38, 39]. Supportive management and allied health that are involved in mentoring care support workers to ensure a positive community environment are particularly imperative. Between organisations, limitations in referral pathways and increased competition between providers leading to fewer collaborations were identified, a sentiment similarly discussed by McLeroy [31]. In contrast, tapping into Dementia Australia and collaborating with research teams to access novel intervention approaches were raised as enabling factors to promoting reablement for people with dementia. Collaboration between care providers and research teams provides an avenue to simultaneously improve the knowledge base and facilitate access to new evidence-based interventions for people living with dementia [45, 46].

The final layer of the social-ecological model, is public policy, which aligns with the pivotal role Australian Government policy has been identified to play in the provision of reablement interventions to support function in people living with dementia in the community. While policy [3] has driven uptake of “reablement” across the community sector (this does not extend to reablement in residential aged care) in an attempt to improve outcomes for people with dementia [47], interviews revealed this to be inconsistent in practice. Interviewees raised barriers around the structure and amount of funding to adequately support people living with dementia. Moreover, limitations remain around what constitutes best reablement practice within the sector. This highlights the need for collaboration between the government and the sector, with dissemination of appropriate tools such as practice guidelines, which have been identified as important facilitators to implementing approaches such as reablement and improving the quality of health care for people living with dementia [47].

This study has several limitations that require consideration. The use of purposive sampling may have introduced bias in the selection of participants. However, purposive sampling is commonly used in qualitative research to maximise efficiency of the process and ensure the inclusion of participants with rich experience with the phenomenon of interest [11, 12]. The sample was taken from three Australian states and is therefore not representative of the entire Australian sector. Despite this, our sampling matrix ensured a diverse sample to increase generalisability across settings. Interviews were conducted with professionals from the aged care sector, however, did not include service users such as people living with dementia and their family members. Thus this research tells ‘one side of the story’ and given there are known barriers to people impacted by dementia using available services [48], future studies are needed to investigate the important perspectives from the service users. Finally, this study solely included providers that deliver services to people living with dementia. Future work should broaden the focus to explore why services may not specifically include people living with dementia and how reablement service delivery may differ between dementia-specific and specifically non-dementia services.

## Conclusions

This study contributes to the knowledge base around the use of reablement interventions for people living with dementia within the Australian aged care sector. Results highlight systemic confusion around the definition of reablement and related concepts, and illustrate the complex interplay of challenges and factors contributing to implementation of reablement in practice, including from policy, the service sector, and clients/service users. Reablement interventions currently provided for people living with dementia in Australia are variable, with apparently limited use of evidence-informed interventions. Future research should illustrate the scope of reablement for people with dementia across different care contexts, i.e., community care, transition care, and residential care. Development of an evidence-informed and freely-accessible resource to support uptake and implementation of reablement interventions for people living with dementia is needed as part of a multifaceted approach that takes into account the varied levels of influence within the Australian aged care sector.

## Supplementary information

**Additional file 1.** Supplement 1.

## Data Availability

The data generated and analysed during the current study are available from the corresponding author on reasonable request.

## Abbreviations

CDPC: Cognitive Decline Partnership Centre
OT: Occupational Therapist
RAS: Regional Assessment Service

## References

[1] Australian Institute of Health and Welfare. Dementia: Australian Institute of Health and Welfare. 2017. http://www.aihw.gov.au/dementia/. Accessed 24 Jan 2017.

[2] Access Economics. Keeping dementia front of mind: incidence and prevalence 2009–2050. Alzheimer's Australia; 2009. https://www.fightdementia.org.au/sites/default/files/20090800_Nat__AE_FullKeepDemFrontMind.pdf. Accessed 24 Jan 2017.

[3] Commonwealth of Australia. Living well at home: CHSP Good Practice Guide. 2015. https://agedcare.health.gov.au/programs-services/commonwealth-home-support-programme/living-well-at-home-chsp-good-practice-guide. Accessed 30 Jan 2018.

[4] Aspinal F, Glasby J, Rostgaard T, Tuntland H, Westendorp RGJ (2016). New horizons: reablement - supporting older people towards independence. Age Ageing.

[5] Poulos CJ, Bayer A, Beaupre L, Clare L, Poulos RG, Wang RH (2017). A comprehensive approach to reablement in dementia. Alzheimers Dement.

[6] Poulos RG, Gresham M, O'Connor CM, Poulos CJ (2018). Bridging the gap: from reablement policy to practice for people with dementia. Alzheimers Dement TRCI.

[7] Forbes D, Forbes SC, Blake CM, Thiessen EJ, Forbes S (2015). Exercise programs for people with dementia. Cochrane Database Syst Rev.

[8] Laver K, Dyer S, Whitehead C, Clemson L, Crotty M (2016). Interventions to delay functional decline in people with dementia: a systematic review of systematic reviews. BMJ Open.

[9] Stokols D (1996). Translating social ecological theory into guidelines for community health promotion. Am J Health Promot.

[10] Guideline Adaptation Committee. Clinical practice guidelines and principals of care for people with dementia. Sydney; 2016.

[11] Marchall MN (1996). Sampling for qualitative research. Fam Pract.

[12] Palinkas LA, Horwitz SM, Green CA, Wisdom JP, Duan N, Hoagwood K (2015). Purposeful sampling for qualitative data collection and analysis in mixed method implementation research. Admin Pol Ment Health.

[13] Cachia M, Millward L (2011). The telephone medium and semi-structured interviews: a complementary fit. Qual Res Organiz Manag.

[14] Sandelowski M (2010). What’s in a name? Qualitative description revisited. Res Nurs Health.

[15] Vaismoradi M, Turunen H, Bondas T (2013). Content analysis and thematic analysis: implications for conducting a qualitative descriptive study. Nurs Health Sci.

[16] Braun V, Clarke V (2006). Using thematic analysis in psychology. Qual Res Psychol.

[17] Harper M, Cole P (2012). Member checking: can benefits be gained similar to group therapy?. Qual Rep.

[18] Carcary M (2009). The research audit trail: enhancing trustworthiness in qualitative inquiry. Electron J Bus Res Methods.

[19] Downe-Wamboldt B (1992). Content analysis: method, applications, and issues. Health Care Women Int.

[20] Thomas DR (2006). A general inductive approach for analysing qualitative evaluation data. Am J Eval.

[21] Graneheim UH, Lundman B (2004). Qualitative content analysis in nursing research: concepts, procedures and measures to achieve trustworthiness. Nurse Educ Today.

[22] Weber RP. Basic content analysis. Beverly Hills, CA: Sage; 1990.

[23] Hsieh HF, Shannon SE (2005). Three approaches to qualitative content analysis. Qual Health Res.

[24] Clemson L, Fiatarone Singh MA, Bundy A, Cumming RG, Manollaras K, O’Loughlin P (2012). Integration of balance and strength training into daily life activity to reduce rate of falls in older people (the LiFE study): randomised parallel trial. BMJ.

[25] Clemson L, Cumming RG, Kendig H, Swann M, Heard R, Taylor K (2004). The effectiveness of a community-based program for reducing the incidence of falls in the elderly: a randomized trial. J Am Geriatr Soc.

[26] Campbell AJ, Robertson MC, Gardner MM, Norton RN, Tilyard MW, Buchner DM (1997). Randomised controlled trial of a general practice program of home based exercise to prevent falls in elderly women. BMJ.

[27] Otago Medical School. Otago exercise programme to prevent falls in older adults: ACC; 2007 https://www.acc.co.nz/assets/injury-prevention/acc1162-otago-exercise-manual.pdf . Accessed 15 Feb 2018.

[28] Spector A, Orrell M, Davies S, Woods B (2001). Can reality orientation be rehabilitated? Development and piloting of an evidence-based programme of cognition-based therapies for people with dementia. Neuropsychol Rehabil.

[29] Spector A, Thorgrimsen L, Woods B, Royan L, Davies S, Butterworth M (2003). Efficacy of an evidence-based cognitive stimulation therapy programme for people with dementia: randomised controlled trial. Br J Psychiatry.

[30] Camp CJ, Judge KS, Bye CA, Fox KM, Bowden J, Bell M (1997). An intergenerational program for persons with dementia using montessori methods. Gerontologist.

[31] McLeroy KR, Bibeau D, Steckler A, Glanz K (1988). An ecological perspective on health promotion programs. Health Educ Q.

[32] Schölmerich VLN, Kawachi I (2016). Translating the socio-ecological perspective into multilevel interventions: gaps between theory and practice. Health Educ Behav.

[33] Brodaty H, Arasaratnam C (2012). Meta-analysis of nonpharmacological interventions for neuropsychiatric symptoms of dementia. Am J Psychiatr.

[34] Swaffer K (2014). Dementia: stigma, language, and dementia-friendly. Dementia.

[35] Gitlin LN, Winter L, Burke J, Chernett N, Dennis MP, Hauck WW (2008). Tailored activities to manage neuropsychiatric behaviors in persons with dementia and reduce caregiver burden: a randomized pilot study. Am J Geriatr Psychiatr.

[36] Hinton L, Franz CE, Reddy G, Flores Y, Kravitz RL, Barker JC (2007). Practice constraints, behavioural problems, and dementia care: primary care physicians’ perspectives. J Gen Intern Med.

[37] Judge KS, Bass DM, Snow AL, Wilson NL, Morgan R, Looma WJ (2011). Partners in dementia care: a care coordination intervention for individuals with dementia and their family caregivers. Gerontologist.

[38] Zwarenstein M, Goldman J, Reeves S. Interprofessional collaboration: effects of practice-based interventions on professional practice and healthcare outcomes. Cochrane Database Syst Rev. 2009;Issue 3. Art. No.:CD000072. 10.1002/14651858.CD000072.pub2 19588316

[39] Grand JHG, Caspar S, MacDonald SWS (2011). Clinical features and multidisciplinary approaches to dementia care. J Multidiscip Healthc.

[40] McKenna HP, Ashton S, Keeney S (2004). Barriers to evidence-based practice in primary care. J Adv Nurs.

[41] Bennett S, Shand S, Liddle J (2011). Occupational therapy practice in Australia with people with dementia: a profile in need of change. Aust Occup Ther J.

[42] Pravikoff DS, Tanner AB, Pierce ST (2005). Readiness of U.S. nurses for evidence-based practice. Am J Nurs.

[43] Hajjaj FM, Salek MS, Basra MKA, Finlay AY (2010). Non-clinical influences on clinical decision-making: a major challenge to evidence-based practice. J R Soc Med.

[44] Cranley LA, Cummings GG, Profetto-McGrath J, Toth F, Estabrooks CA. Facilitation roles and characteristics associated with research use by healthcare professionals: a scoping review. BMJ Open. 2017:e014384. 10.1136/bmjopen-2016-014384 PMC5724142 28801388

[45] Gitlin LN, Marx K, Stanley IH, Hodgson N (2015). Translating evidence-based dementia caregiving interventions into practice: state-of-the-science and next steps. Gerontologist.

[46] Morris ZS, Wooding S, Grant J (2011). The answer is 17 years, what is the question: understanding time lags in translational research. J R Soc Med.

[47] Dougherty D, Conway PH (2008). The “3T’s” road map to transform US health care: the “how” of high-quality care. JAMA.

[48] Brodaty H, Thomson C, Thompson C, Fine M (2005). Why caregivers of people with dementia and memory loss don't use services. Intl J Geriatr Psych.

