# Supplementary material for: Understanding in the Australian aged care sector of reablement interventions for people living with dementia: a qualitative content analysis
Source: BMC Health Serv Res. 2020 Feb 24;20:140. doi: 10.1186/s12913-020-4977-1 (PMC7041110; doi:10.1186/s12913-020-4977-1)
Supplement: Supplementary file 1 — Additional file 1. Supplement 1. [file 12913_2020_4977_MOESM1_ESM.pdf]

HammondCare
An independent Christian charity

COGNITIVE DECLINE PARTNERSHIP CENTRE

UNSW SYDNEY

SURVEY OVERVIEW

DEVELOPMENT OF EVIDENCED BASED DEMENTIA REABLEMENT GUIDELINES AND PROGRAMS: SURVEY OF AGED CARE PROVIDERS

Professor Chris Poulos

This is an overview of the topics we would like to cover with the representative from your organisation.

Questions about the organisation and the interviewee:

- How would your organisation be best described (i.e. is it a not-for-profit, for-profit, or a government organisation)?
- How many states does your organisation operate in?
- Does your organisation provide residential aged care services?
- Does your organisation provide community aged care services?
- Would you say that your organisation focuses on general aged care services or is the organisation more a speciality provider of dementia services?
- What does your role within the organisation involve?

Terminology and roles:

- Discuss terms such as 'reablement', 'restorative care', 'functional ability'.
- Discuss the roles in your organisation that are involved in providing reablement-type services for people with mild to moderate dementia

Provision of services that promote function for people with dementia:

- Discuss any programs provided by your organisation that could be classed as 'reabling' for people with mild to moderate dementia

Barriers and facilitators:

- Discuss factors which may support or hinder the provision of 'reablement-type' services to people with mild to moderate dementia
